# Average acceleration and intensity gradient of 9–11-year-old rural and urban Kenyan school-going children and associations with cardiorespiratory fitness and BMI: The Kenya-LINX project

**DOI:** 10.1371/journal.pone.0329173

**Published:** 2025-08-04

**Authors:** Stanley K. Kinuthia, Gareth Stratton, Lucy J. Wachira, Victor O. Okoth, George Evans Owino, Sophie Ochola, Festus Kiplamai, Vincent Onywera, Nils Swindell

**Affiliations:** 1 Department of Physical Education, Exercise and Sports Science, Kenyatta University, Nairobi, Kenya; 2 Applied Sports, Technology, Exercise and Medicine (A-STEM) Research Centre, Swansea University, Swansea, United Kingdom; 3 Department of Environmental Science and Education, Kenyatta University, Nairobi, Kenya; 4 African Population and Health Research Center, Kitisuru, Manga Close, Nairobi, Kenya; 5 Department of Food, Nutrition and Dietetics, Kenyatta University, Nairobi, Kenya; 6 Division of Research, Innovation and Outreach, KCA University, Nairobi, Kenya; eCampus University: Universita degli Studi eCampus, ITALY

## Abstract

**Background:**

Physical activity (PA) is crucial for children’s health, cardiorespiratory fitness (CRF) and weight status. However, research on the PA profiles of Kenyan children, especially between rural and urban areas, is limited.

**Method:**

This study examined the PA profiles of 537 school-aged children (51.6% girls, 9–11-year-olds) from Nairobi City County, Kenya (urban setting), and Kitui County, Kenya (rural setting), using stratified multistage random cluster sampling. Participants wore an accelerometer (Axivity AX3) on their non-dominant wrist for 24 hours a day over seven days. Raw accelerations were extracted and averaged over a 5-s epoch (AvgAcc) to estimate PA volume. Intensity gradient (IG) was calculated as a standardised metric of PA intensity. Participants’ CRF was assessed by the 20-metre multistage fitness test. Anthropometric measurements (mass and stature) were taken to compute BMI z-scores.

**Results:**

PA metrics varied by sex, weight, and CRF. Boys had higher AvgAcc (p < .001) and IG (p < .001) than girls. Healthy weight participants differed significantly in AvgAcc from overweight (p = .001) and obese (p = .001) groups and in IG from overweight (p = .039) and obese (p = .003). Participants with sufficient CRF and insufficient CRF differed significantly in AvgAcc (p < .001) and IG (p < .001). AvgAcc was negatively associated with BMI z-scores (β = −0.02, p < .001) and positively associated with CRF (β = 0.21, p < .001), independent of IG and other covariates. IG showed a significant negative association with BMI z-scores (β = −1.27, p = 0.003) and a significant positive association with CRF (β = 18.93, p < .001), dependent on AvgAcc.

**Conclusions:**

This study introduces AvgAcc and IG metrics among Kenyan children. Urban children accumulate less PA volume and exhibit an inferior intensity profile, which is reflected in important health indicators (BMI and CRF). These findings will inform policy as well as targeted interventions to enhance children’s health in diverse contexts.

## Introduction

Physical activity (PA) positively impacts children’s physical, psychosocial, and cognitive well-being [1]). The key benefits include reduced risks of obesity (OB) ( [2]), diabetes, hypertension [3], cardiovascular disease [4], and all-cause mortality [5], along with improved mental health and academic achievement [6]. Despite these benefits, 50% of children in Kenya do not meet the global guidelines of 60 minutes of moderate-vigorous PA (MVPA) [7]. This figure rises to 87.4% in urban areas [8], highlighting disparities in children’s PA and health. This trend of insufficient PA is a widespread issue in Sub-Saharan Africa, notably in urban settings in countries like Kenya [9], South Africa [10], Senegal [11], and Uganda [12]. Kenyan rural areas offer more opportunities for PA during school hours, resulting in higher levels of daily PA and improved adherence to movement guidelines. On the other hand, children in urban areas exhibit higher levels of sedentary behaviour, lower levels of PA, and poor CRF [13–16]. Globally, girls are more sedentary than boys (85% versus 77.6%, respectively), with 4 out of every 5 adolescents aged 11–17 failing to meet PA recommendations and 81% lacking sufficient PA [17]. These statistics emphasise the compelling need to promote PA through the design of sustainable programmes and interventions as a public health priority, particularly for children.

Studies utilising device-based measures of PA in Sub-Saharan Africa are scarce, with existing studies primarily using a cut-point analysis approach [8,10–12,15]. Using cut points has several limitations, as it often misses incidental PA and only provides daily PA information in brief bouts, which can be prone to inaccuracies [18]. Moreover, interpretations are complicated by the close relationship between time spent below cut-points and above cut-points, and different cut-points applied to the data yield varying outcomes/results [19,20]. As the cut point rises, a growing percentage of the cohort may register a zero-activity score, especially in vigorous PA, which obscures the amount of activity accrued [4]. This underreporting makes it challenging to draw appropriate and accurate conclusions about the cardiometabolic health of children and hampers the design of effective interventions [21]. The dependence on population- and protocol-specific calibration techniques further limits the replication of other studies and the application of findings for surveillance and intervention analysis [22].

In contrast to cut-point pathways, combining IG with AvgAcc accurately describes an individual’s 24-hour physical behaviour profile, giving a comprehensive understanding of PA instead of relying on small chunks of cut-point-derived PA [19,22–24]. AvgAcc correlates with CRF and MVPA, while IG is positively associated with CRF among school children in the UK [25]. Higher-intensity PA is consistently related to strong health indicators compared to lower -intensity PA [26]. Using AvgAcc and IG minimises errors inherent in employing physical behaviour outcomes, as both metrics are generated from directly measured acceleration [27]. These metrics are procedure- and demographic-independent, easing comparisons across studies [27]. While the importance of PA volume (PAV) is acknowledged, intensity plays a more essential role in promoting health in children [24]. Most device-based research work deploying these metrics has been conducted among European children [19,22–24]. Understanding how AvgAcc and IG relate to the health outcomes in children, particularly regarding weight status and CRF in diverse contexts in Kenya, remains largely unexplored.

Child demographics such as sex [28], age [29], and obesity status [30] are consistently connected to PA. Generally, boys exhibit favourable PAV and intensity profiles than girls [25]. Cardiorespiratory fitness is a key determinant of total weekly PA minutes in children [31,32]. Additionally, MVPA is associated with higher CRF levels and healthier weight in children [33], highlighting its role in enhancing health outcomes in children. Children with low PA levels are more likely to be overweight and have lower CRF, which is associated with a poorer metabolic profile. In contrast, active children maintain a healthy weight and higher CRF [34,35]. Kenya requires improved infrastructure and school-based initiatives to increase PA among children, but there is insufficient data to back these efforts. Thus, the associations between PAV and intensity with health outcomes like weight status and CRF will inform the design of health interventions, translating metrics into public-friendly health promotion messages tailored for specific populations.

This paper aligns with the Global Action Plan on Physical Activity (GAPPA) by the WHO, which aims to reduce insufficient PA among adolescents (aged 11–17 years) by 15% by 2030 [36]. The timing of this research is also crucial, as the Kenyan government is implementing the new Competency-Based Curriculum (CBC) in primary schools, with emphasis on physical education (PE) and sports [37]. This shows the government’s appreciation of the significance of PA and physical fitness to the health, academic performance, and well-being of the children, recognizing them as vital resources for the nation’s future.

This study combines the Health Belief Model (HBM) [38] with the General Systems Theory (GST) [39] to explore the relationships between PA, CRF, and weight status among children in Kenya. The HBM provides an understanding of how personal perceptions impact PA behaviours, while the GST emphasises how environmental factors, such as living in urban or rural areas, affect health outcomes.

Therefore, this study sought to i) utilise AvgAcc and IG to compare PA profiles of girls and boys from urban and rural Kenya and ii) examine the associations between PA volume and intensity with CRF and weight status.

## Materials and methods

### Subjects and settings

This study emanated from larger collaborative research between researchers from Kenya and Swansea called “Kenya Healthy Diet and Active Lifestyle Infrastructure for the NeXt Generation (Kenya-LINX),” which was based on the SportsLinx project [40]. Two study sites were chosen to represent Kenya’s urban (Nairobi City County) and rural (Kitui County) settings, capturing two distinctly different environmental and lifestyle characteristics of the Kenyan children. A total of 537 children (boys = 260 (48.42%), 9–11 years of age (11.1 ± 0.9 years) who attended private and public day mixed primary schools (3rd to 5th grades) participated in the study between 4-03-2021 and 28-01-2022. The study recruited 8 rural (Nairobi City County) and 8 urban (Kitui County) schools. The Kenyatta University Institutional Ethical Review Committee [Reference number: PKU2106/11254; date of approval: 04/05/2020, renewed on 02/07/2021] and the National Commission for Science, Technology, and Innovation (NACOSTI) [License No: NACOSTI/P/20/5030] granted the approvals for the study. Additionally, relevant approvals were granted by the county and sub-county offices within the two counties. Nairobi [Ref: GL/NC/142. VOL VI/345) and Kitui (Ref. No: KTIC/ED/Res/Vol. l/22/113]. The collection of data took place between March 2021 and January 2022. Prior to the commencement of the research, written legal guardian/carer/parent informed consent and child assent were obtained.

This study utilised the HBM and GST to structure its approach, identifying contextual variables such as age, sex, and area of residence as system inputs and PA metrics (AvgAcc and IG) as throughputs within the child’s health system. Accelerometers were used to objectively assess PA in urban and rural environments, examining how individual perceptions and environmental elements influence behaviours, ultimately impacting CRF and BMI z-scores as health outcomes.

## Measures

### Weight status

Stature (to the nearest 0.1 centimetre) and mass (to the nearest 0.1 kilogram) of barefoot subjects in light clothing were measured using a portable stadiometer (SECA 217, Hamburg, Germany) and a portable digital scale (SECA 813, Hamburg, Germany), respectively. By dividing each subject’s weight by their squared height, the body mass index (BMI) was determined. The resulting BMI z-scores were then used to classify participants as below a healthy weight, HW, OW, or, OB based on the International Obesity Task Force BMI cutoffs [41].

### Cardiorespiratory fitness

The 20-m multistage shuttle run test (20m MSFT), widely utilized among participants of the same age [42,43], was used to estimate children’s CRF. Cut points classifying children as fit and unfit were assigned according to the number of shuttles completed (boys ≥33 shuttles, girls ≥25 shuttles), as these thresholds reflect cardiometabolic risk scores in children of this age group [44]. The test exhibits high reliability [45] and moderate-to-high criteria-related validity [46].

### Physical activity

Participants were provided with oral and written instructions on the usage of the accelerometer, specifically to wear an initialised Axivity AX3 (Axivity Ltd., Newcastle, UK) accelerometer on their non-dominant wrist for 24 hours a day for seven days. The Axivity AX3 accelerometer is a proven tool for use with children, effectively identifying PA, sedentary behaviour, and posture in this demographic [47,48]. It reported accuracy rates of 74–96% for detecting activity intensity in children aged 6–16 years [47]. This device has been utilised in large-scale studies, including over 900 children aged 9–13 years in Denmark [49] and in the UK Biobank study [50]. Its reliability in capturing movement data is well- supported [51–55]. It has been validated against established measures of PA energy expenditure [56]. The device’s small size, water resistance, long lasting battery, and high-resolution capabilities enhance user compliance, making it particularly suitable for use with children [18,56–58].

Accelerations were recorded at 100 Hz with a dynamic range of ± 8 g. OmGui (Version 1.0.0.43, Open Movement, Newcastle University, UK) was used to download data that were converted to.csv format for data processing after being saved in raw format as.cwa files. All data were processed in R (http://cran.r-project.org) using GGIR v2.3.0 [59]. Data processing included auto-calibration with reference to local gravity [60], detection of repeatedly high abnormal values and non‐wear detection. The raw triaxial acceleration values were converted into a single omnidirectional measure of acceleration known as Euclidean Norm minus 1 g (ENMO). This was done by subtracting 1 g from the raw accelerations of the three axes, which stands for the value of gravity $(\text{ENMO}\;=\sqrt{}\;(x^{2}\;+y^{2}\;+\;z^{2}-\;1$. The ENMO values were expressed in milli-gravitational units (mg) and averaged over epochs of 5 seconds. Negative values were rounded to zero.

Accelerometer non-wear periods were computed for 60-minute windows with a 15-minute sliding window using the standard deviation (SD) and value range of the accelerations at each axis as the basis [61]. The time window was deemed non-wear if the SD for at least 2 of the 3 axes was less than 13 mg or the value range was less than 50 mg. The broadly used minimal threshold for measuring the 24-h movement behaviours of 16 h/day was applied [62]. Participants’ Axivity files were removed from the analysis if they had post-calibration errors greater than 10 mg (0. 01 g) [59,61] and if the number of wear days that qualified as having a duration of at least 16 hours per day was fewer than 3 days (having a duration of at least 4 days including 1 weekend day) [62,63] or wear data was absent for each of the 24-hour cycles’ 15-minute windows.

Out of the total 537 children initially recruited for the study, 56 participants (10.4%) were excluded, resulting in a final analytical accelerometry sample of 481 participants. Among participants who met this wear time criteria, an average of 0.9 ± 3.4% and 1.4 ± 3.4% of each 24 h was identified as non-wear time for Kitui County (rural) and Nairobi City County (urban), respectively, with no significant difference between the counties (t (425.7) = −1.5, p < 0.134). Epochs identified as non-wear time were imputed at the epoch level based on the individual’s mean value from the same time slot in the day of other valid days in the recording.

The complete 24-hour cycle (1440 minutes) for all 481 participants was used as the basis for their outcome variables. Total PA (AvgAcc) across the day (ENMO, mg) and IG were used to express the daily PA. The procedure outlined by Rowlands and others was used to calculate the IG metric in GGIR and was represented’ by the ‘AD_IG’ variable [20]. The relationship between the intensity log values (i.e., incremental intensity bins such as those for 0-25 mg, 25-50 mg, etc.) and time (i.e., accumulated time in every intensity bin) is consistently negative, representing the amount of time dropped into progressively higher intensity bins. The linear regression equation’s constant, the individuals’ IG over a 24-hour period, and the R^2^ value, which shows how well the linear model fits the data, were generated for every participant.

### Statistical analysis

Descriptive statistics were calculated to present continuous variables (BMI z-score, completed laps, age, AvgAcc, and IG) using mean (SD) and categorical variables (sex, CRF status, and weight status) as absolute frequencies and percentages. Linear mixed models with random intercepts were used to compare the dependent variables (IG and AvgAcc) by sex, weight status, and CRF, accounting for school-level clustering. Distinct models were developed to assess the association between PA metrics and health indicators, specifically CRF and BMI z-score. Model 1 only had the PA metric (i.e., AvgAcc or IG). Model 2 analysis was adjusted for age, sex, and area of residence, while Model 3 was further adjusted for BMI z-score (just for CRF) and included both AvgAcc and IG (alternate activity metric) to test whether associations were independent. Moreover, model 4 included the interaction effect between sex and IG. Continuous variables (PA metrics) were centred before entry into linear mixed model and regression analyses; results were reported with a 95% CI. The variance inflation factor (VIF) analysis was used to assess multicollinearity (see data in S5 Table). The outcomes of the VIF values for predictor variables were < 5 indicating multicollinearity was not an issue [64]. Unpaired t-tests and chi-square tests were used to compare the included and excluded participants for continuous and categorical variables with missing data, respectively. The resulting p-values were non-significant, indicating that the data were missing at random. The proportion of missing values excluded for each variable varied between 0.9% and 3.7%, which is within acceptable limits for excluding data [65]. For group comparisons (urban vs. rural, male vs. female), analyses were conducted on the available data for specific variables with occasional missing values. In cases where anthropometric measurements were missing, listwise deletion was applied. All the data analyses were conducted using IBM SPSS version 28 software, with statistical significance determined at p < .05.

## Results

### Descriptive characteristics of the participants

Descriptive statistics are presented in Tables 1 and 2. A sample of 537 children (207 rural, 330 urban) revealed significant disparities in demographics and health metrics (p < 0.05). Rural children were older (11.23 vs. 10.95 years) but shorter (137.0 cm vs. 141.3 cm). Urban children were heavier (36.86 kg vs. 30.21 kg) and had a higher BMI (18.42 vs. 15.96 kg/m²). Urban areas showed a higher prevalence of HW (63.5% vs. 50.3%), while rural areas had more BHW children (42.1% vs. 14.6%). Only 18.7% of rural and 8.8% of urban children had sufficient CRF. Rural children exhibited a higher AvgAcc (54.92 mg vs. 48.36 mg), and a slightly superior IG (−2.04 vs. −2.09).

**Table 1:** Continuous variables – rural vs. urban characteristics.

Variable	All (n = 537) Mean (SD)	Rural (n = 207) Mean (SD)	Urban (n = 330) Mean (SD)	t-value	P-value
Age (years)	11.05 (0.87)	11.23 (0.98)	10.95 (0.78)	−3.66	<.001^*^
**Body size measures**					
Height (cm)	139.0 (9.5)	137.0 (7.21)	141.3 (10.34)	5.04	<.001^*^
Mass (kg)	34.38 (8.9)	30.21 (6.98)	36.86 (8.99)	8.85	<.001^*^
BMI (kg/m^2^)	17.51 (3.8)	15.96 (2.62)	18.42 (4.01)	7.71	<.001^*^
BMI z-score	−0.21 (1.47)	−0.98 (1.27)	0.23 (1.40)	9.90	<.001^*^
**Cardiorespiratory fitness**					
Laps completed	17.39 (10.20)	19.63 (11.53)	16.05 (9.06)	−3.95	<.001^*^
**24-hours PA profile-all days**					
Average acceleration (mg)	50.84 (14.52)	54.92 (15.29)	48.36 (13.44)	−5.02	<.001^*^
Intensity Gradient	−2.07 (0.16)	−2.04 (0.15)	−2.09 (0.16)	−2.68	0.008^*^
Constant/Intercept	13.00 (0.67)	12.95 (0.65)	13.04 (0.68)	1.30	0.196
Explained variance (*R*^2^)	0.93 (0.01)	0.93 (0.02)	0.93 (0.01)	4.34	<.001^*^

* p < 0.05, compared rural and urban characteristics. Abbreviations: BMI = body mass index; SD = standard deviation; PA = physical activity.

**Table 2:** Categorical variables – rural vs. urban characteristics.

Variable	All (n = 537)	Rural (n = 207) n (%)	Urban (n = 330) n (%)	chi-squared	P-value
**Sex**					
Boys	260 (48.42%)	98 (47.30%)	162 (49.09%)	15.68	0.003^*^
Girls	277 (51.58%)	109 (52.7%)	168 (50.91%)		
**Weight status categories**					
Healthy weight	307 (58.6%)	98 (50.3%)	209 (63.5%)	60.61	<.001^*^
Below healthy weight	132 (25.2%)	84 (42.1%)	48 (14.6%)		
Overweight	70 (13.4%)	12 (6.2%)	58 (17.6%)		
Obese	15 (2.9%)	1 (0.5%)	14 (4.3%)		
**Cardiorespiratory fitness**					
Sufficient CRF	66 (12.52%)	37 (18.7%)	29 (8.8%)	40.49	<.001^*^
Insufficient CRF	461 (87.48%)	161 (81.3%)	300 (91.2%)		

* p < 0.05, compared rural and urban characteristics. Abbreviations: CRF = cardiorespiratory fitness. χ2 = chi-square test.

Note: sex and CRF differences were derived using fishers’ exact test.

### PA differences in average acceleration and intensity gradient between sex, weight status, and cardiorespiratory fitness status groups

Significant differences in PA metrics between boys and girls were observed for AvgAcc (p < .001) and IG (p < .001) (Table 3). For AvgAcc, significant differences were observed between HW and OW participants (p = 0.001) and OB participants (p = 0.001) and those with sufficient and insufficient CRF (p < .001). Regarding IG, significant differences were observed between HW and OW participants (p = 0.039) and OB participants (p = 0.003), as well as between those with sufficient and insufficient CRF (p < .001). There were no significant differences for AvgAcc and IG among underweight children.

**Table 3:** Between-group differences (24-hour activity profile) in average acceleration and intensity gradient (n = 481).

	Average acceleration (m*g*)	P value	Intensity Gradient	P value
	Mean (SD)		Mean (SD)	
**Sex**				
Boys (reference)	56.13 (15.12)	<.001^*^	−1.99 (0.13)	<.001^*^
Girls	46.09 (11.88)		−2.14 (0.14)	
**Weight status**				
Healthy weight (reference)	51.45 (14.39)		−2.06 (0.16)	
Overweight	43.28 (9.86)	<.001^*^	−2.13 (0.15)	0.039^*^
Obese	38.65 (7.08)	<.001^*^	−2.19 (0.15)	0.003^*^
Below healthy weight	55.98 (14.79)	0.118	−2.04 (0.13)	0.346
**CRF**				
Sufficient CRF (reference)	60.34 (13.93)	<.001^*^	−1.98 (0.12)	<.001^*^
Insufficient CRF	49.56 (13.93)		−2.08 (0.15)	


* *p* < 0.05, compared between-group differences in average acceleration and intensity gradient.

Abbreviations: SD = standard deviation, CRF = cardiorespiratory fitness.

### Association of PA metrics (average acceleration and intensity gradient) with health indicators (CRF and BMI z-score)

Table 4 displays the findings from the models examining the associations between AvgAcc and IG with health indicators (CRF and BMI z-score). Significant effects for either AvgAcc or IG indicated which (volume or intensity) had a stronger association with a given health indicator, i.e., CRF and BMI z-score, highlighting the importance of understanding how different aspects of PA contribute to these health outcomes. For the first (unadjusted), second (adjusted), and third (adjusted) models, respectively, AvgAcc was negatively (favourably) significantly associated with BMI z-score and positively significantly associated with CRF. In the third (adjusted) model, AvgAcc was negatively significantly associated with BMI z-score. However, when IG was included, this association was no longer significant, indicating that IG did not have an independent effect on BMI z-score after accounting for total PAV (AvgAcc) (detailed results are in S1 and S2 Tables). Both in the unadjusted (Model 1) and confounding factor-adjusted (Model 2), IG showed a significant positive association with CRF. Conversely, IG showed a significant negative association with BMI z-score in both unadjusted and adjusted models (models 1 and 2, respectively) (see data in S3 and S4 Tables).

**Table 4:** Cross-sectional associations between 24-hour physical activity profile metrics and health indicators in children (n = 481).

Variable	Model 1	Model 2	Model 3	Model 4
	*β (95% CI) p-value (VIF)*	*β (95% CI) p-value (VIF)*	*β (95% CI) p-value (VIF)*	*β (95% CI) p-value (VIF)*
**CRF**				
AvgAcc (mg)	0.21 (0.15, 0.27) <0.001^*^ (1.000)	0.12 (0.06, 0.19) <0.001^*^ (1.011)	0.10 (0.02, 0.17) 0.018^*^ (1.033)	0.10 (0.02, 0.18) 0.011^*^ (1.034)
IG	18.93 (13.27, 24.59) <0.001^*^ (1.000)	9.81 (3.45, 16.17) 0.003^*^ (1.011)	4.79 (−2.81, 12.40) 0.216 (1.042)	1.41 (−7.57, 10.39) 0.757 (1.189)
**BMI z-score**				
AvgAcc (mg)	−0.02 (−0.03, −0.01) <0.001^*^ (1.000)	−0.02 (−0.03, −0.01) <0.001^*^ (1.011)	−0.02 (−0.03, −0.01) 0.001^*^ (1.033)	
IG	−1.27 (−2.11, −0.44) 0.003^*^ (1.000)	−1.52 (−2.44, −0.60) 0.001^*^ (1.011)	−0.44 (−1.55, 0.66) 0.431 (1.042)	

* p < 0.05, association between AvgAcc and IG with CRF and BMI z-score.

Statistical tests: Linear mixed model analysis was used for all models (t-tests for individual coefficients).

Abbreviations: β = Unstandardized regression coefficient (β values represent the change in health indicator for a 1-unit change in the PA metric), CI = confidence interval, VIF = variance inflation factor, CRF = cardiorespiratory fitness, AvgAcc = average acceleration, IG = intensity gradient, BMI z-score = BMI expressed in z-scores.

Model specifications:

Model 1: Adjusted for clustering at the school level.

Model 2: Additionally adjusted for sex, area, and age.

Model 3: Further adjusted for weight status (but only for CRF) and alternate physical activity metric (AvgAcc and IG).

Model 4: Included the interaction effect between IG and sex [M].

Note: Scores were centered before entering the analysis

## Discussion

This study, the first in Kenya to compare device-measured PA metrics (AvgAcc and IG) between primary school children in urban and rural settings, interprets its findings using the combined model of the HBM and GST. This approach provides a deeper understanding of the factors affecting children’s CRF and weight status. Our findings, viewed through GST, show how system inputs (urban/rural residency and sex) and throughputs (PA patterns) interact to produce outputs (health indicators such as CRF and weight status). The HBM provides additional insights into the observed differences in PA and health outcomes.

The study found that children attained overall AvgAcc values of 50.84 mg and mean IG values of −2.07. These findings are comparable and align with previous studies involving school children aged 9–10 years (45.4 mg and −1.96) [25], 8−12 years (44.2 mg and −1.99) [66], girls aged 8−14 (36.3 mg and −2.47) [19], and 8–13 years (41.6 mg and −2.09) [67]. Higher AvgAcc indicates greater PAV linked to better health outcomes [68,69], while lower IG indicates poorer intensity distribution associated with poorer health outcomes [66,70]. These metrics highlight the importance of both the volume and intensity of PA, suggesting that exercise and PA guidelines should promote both higher PAV (AvgAcc) and higher intensity activities (IG) to optimise health outcomes [66,70,71].

Children from rural areas demonstrated higher AvgAcc and IG levels compared to their peers from urban areas. This aligns with previous studies in Kenya and Sub-Saharan Africa that have used cut points showing higher PA levels among rural children than their urban peers [14,72,73], likely due to greater reliance on active transportation, such as walking or cycling, to and from school and for daily errands, as well as more opportunities for outdoor play offered by rural environments [72,73]. These serve as beneficial system inputs (GST) that foster higher PA throughputs. In contrast, children living in urban environments frequently depend on motorised transport, which limits opportunities for incidental PA and contributes to increased sedentary behaviours. In sum, these act as inputs that lead to lower PA throughputs and potentially higher BMI z-scores [13,74]. These observed differences reflect both individual barriers to PA and broader systemic influences, that lead to variations in health outcomes. These inequalities further underscore the need to promote active commuting and create environments that encourage PA, especially in urban areas.

While this study did not directly address socioeconomic factors, existing literature indicates a connection between urban living, higher economic status, and lower PA levels in children [75–77]. For example, 50% of urban children in Kenya exceed recommended screen time limits, which may contribute to increased sedentary behaviour and unhealthy weight trends [78]. The present study found that over half of urban children were classified as HW, while more than a quarter of rural children were below healthy weight. The difference in weight outcomes likely stems from unequal access to healthy food. Fresh produce is often more accessible in urban areas, while rural areas frequently struggle with affordable, nutrient-dense options [79].

The findings reveal that Kenyan children generally have low CRF levels, with only a small proportion meeting sufficient thresholds. This discovery stresses the urgent need for school-based initiatives to improve CRF. A higher proportion of children in rural areas than urban areas exhibit adequate CRF, likely due to better access to outdoor spaces and more habitual physical tasks after school, which are less common in urban environments, as concluded by previous studies [13,72,76]. The low overall CRF levels observed underscore a systemic need for interventions to improve PA throughput in Kenyan children, particularly in urban areas. These findings underscore the significance of promoting PA within schools. They also emphasise the necessity of raising awareness about PA among parents and within the community to enhance children’s overall fitness levels.

The study noted significant differences in PAV and intensity based on CRF, weight status, and sex. Boys, HW children, and those with adequate CRF exhibited higher AvgAcc and better IG profiles. In contrast, below healthy weight children showed no significant differences in PA between urban and rural children. These findings are consistent with previous research indicating that boys typically engage in more MVPA than girls, potentially due to physiological differences [80,81] and social norms encouraging boys and/or discouraging girls from prioritising PA [17]. The higher AvgAcc and better IG profiles in HW children compared to OW and OB children support literature showing lower PA levels are consistently evident in higher weight categories [82,83].

Demographic differences in AvgAcc and IG highlight how initial system inputs (GST) shape children’s PA profiles. Disparities between boys and girls arise from societal expectations and available opportunities [13,17,73], influencing health outcomes. Variations across different weight categories and CRF levels demonstrate the interaction between PA throughput and health metrics, creating a feedback loop that affects children’s overall health and perceptions of health risks.

Previous research in Kenya reveals urban children have better nutritional status but higher body fat [16,79], while rural children have more opportunities for PA, are more active during school hours, and have higher CRF levels [13,16]. Additionally, children in rural areas more closely adhere to 24-hour movement guidelines, particularly in sleep and PA [13]. These results demonstrate the disparities between urban and rural settings in shaping children’s health and activity profiles. Therefore, to effectively enhance PA and CRF, interventions should focus on addressing factors that affect individuals’ perceptions of their vulnerability to the risks of inadequate activity, the benefits of increased fitness levels, and barriers such as lack of safe spaces and time constraints, all of which are central components of the HBM.

The study found a significant negative association between AvgAcc and BMI z-scores independent of IG and potential correlates such as age, sex, and area (rural and urban). This indicates that higher total activity volume correlates with healthier weight, suggesting that even low-intensity activities contribute to calorie expenditure and weight management. This finding is in line with previous research in European studies [24,25,66]. Additionally, AvgAcc was positively associated with CRF, reinforcing the importance of total activity volume in promoting healthy weight and CRF and ultimately overall health of children. These observed associations demonstrate how throughput elements (AvgAcc and IG) are linked to outputs (health indicators), as predicted using GST.

The study also found that IG was negatively associated with BMI z-scores independent of potential correlates and dependent on AvgAcc, which partially supports earlier findings [19,25,66]. This contrasts with studies emphasising that vigorous activity is crucial for preventing excessive weight gain in children [84]. In a study involving adolescents aged 11–14 years, including IG in the analysis diminished the relationship between AvgAcc and CRF, highlighting the complex interplay between activity volume, intensity, and fitness. The diminishing association between IG and BMI z-score by AvgAcc highlights the interaction of various throughput elements within the system. In this context, the overall volume of activity (AvgAcc) takes precedence over the intensity of activity (IG) in shaping certain system outputs (BMI z-score as a health outcome).

The present study also found a positive association between IG and CRF independent of correlates such as sex, age, area (rural and urban), and BMI z-score. However, this association was attenuated by AvgAcc, indicating that both PAV and intensity contribute to CRF. This differs from previous studies which suggest that the association between IG and CRF is independent of AvgAcc [19,25,66]. The attenuation implies that CRF benefits from activities of high intensity, while PA volume also plays a crucial role. The findings imply that children with higher baseline fitness benefit more from activities of higher intensity, while those with lower fitness gain from both increased PA volume and intensity [85]. This highlights the need for personalised activity recommendations to optimise fitness outcomes for children.

The study has several limitations. For example, the absence of socioeconomic data limits the comprehension of how income affects PA, BMI, and CRF. The study’s cross-sectional design restricts the ability to conclude causal relationships between CRF BMI z-scores and PA metrics; longitudinal research is necessary for a more in-depth understanding. Additionally, even after controlling for BMI z-score, the absence of dietary or nutritional data might have impacted weight outcomes. Furthermore, the study did not account for the effects of climatic conditions or seasonal variations on the activity patterns of children in Kenya. Future studies should adopt longitudinal designs with repeated measures across various seasons to investigate these influences. Nonetheless, the use of a large sample from both rural and urban schools is a notable strength of the study, allowing for urban and rural comparisons. Furthermore, the use of linear mixed models that account for school-level clustering and include covariates that influence activity levels. The use of novel metrics (IG and AvgAcc) that capture both volume and intensity of PA over 24 hours are a further strength of the study, moving away from a cut-point approach to PA measurement that supports comparisons across populations and studies.

## Conclusions

This study is the first to evaluate IG and AvgAcc metrics in primary school-aged Kenyan children wearing wrist-worn Axivity monitors. Integrating GST with HBM is crucial for successful health interventions, improving public health strategies by considering the contextual elements that affect perceived barriers and benefits. Key findings indicate that boys have higher AvgAcc and IG than girls, whereas HW children engage in more PA than their OW or OB peers. Total PA volume, as measured by AvgAcc, is crucial for both CRF and BMI z-scores, linking increased activity volume to improved CRF and lower BMI z-scores. Although IG positively impacts CRF, its association with BMI z-scores is reduced by AvgAcc. By addressing gender disparities, focusing on total PA volume while gradually incorporating intensity (IG) to build fitness, and tailoring interventions for OW/OB children, these policies can foster healthier, more active lifestyles among Kenyan children, improving fitness and weight outcomes.

## Supporting information

S1 Table Association between cardiorespiratory fitness and average acceleration. (DOCX)

S2 Table Association between BMI z-score and average acceleration. (DOCX)

S3 Table Association between cardiorespiratory fitness and intensity gradient. (DOCX)

S4 Table Association between BMI z-score and intensity gradient. (DOCX)

S5 Table VIF values for all models (Cardiorespiratory fitness and BMI z-score). (DOCX)

S6 Table Inclusivity in global research. (DOCX)
