# Supplementary material for: Average acceleration and intensity gradient of 9–11-year-old rural and urban Kenyan school-going children and associations with cardiorespiratory fitness and BMI: The Kenya-LINX project
Source: PLoS One. 2025 Aug 4;20(8):e0329173. doi: 10.1371/journal.pone.0329173 (PMC12321071; doi:10.1371/journal.pone.0329173)
Supplement: S1 Table — (DOCX) [file pone.0329173.s001.docx]

S1 Table. Association between cardiorespiratory fitness and average acceleration

|  | | | | | | | | | | | | |
| --- | --- | --- | --- | --- | --- | --- | --- | --- | --- | --- | --- | --- |
|  | *Model 1* | | | *Model 2* | | | *Model 3* | | | *Model 4* | | |
| **Predictors** | **Estimates** | **CI** | **p** | **Estimates** | **CI** | **p** | **Estimates** | **CI** | **p** | **Estimates** | **CI** | **p** |
| **(Intercept)** | 6.71 | 3.12 – 10.31 | **<0.001** | 1.59 | -9.83 – 13.02 | 0.784 | 12.69 | -8.29 – 33.67 | 0.235 | 7.94 | -15.10 – 30.98 | 0.499 |
| **AD mean ENMO mg 0 24hr** | 0.21 | 0.15 – 0.27 | **<0.001** | 0.12 | 0.06 – 0.19 | **<0.001** | 0.10 | 0.02 – 0.17 | **0.018** | 0.10 | 0.02 – 0.18 | **0.011** |
| **Sex [M]** |  |  |  | 4.85 | 3.17 – 6.53 | **<0.001** | 4.44 | 2.64 – 6.24 | **<0.001** | 23.60 | -0.66 – 47.85 | 0.056 |
| **County [N]** |  |  |  | -1.32 | -4.41 – 1.76 | 0.400 | -1.29 | -4.38 – 1.80 | 0.412 | -2.66 | -6.01 – 0.69 | 0.119 |
| **Age** |  |  |  | 0.70 | -0.25 – 1.66 | 0.149 | 0.74 | -0.22 – 1.70 | 0.129 | 0.71 | -0.24 – 1.66 | 0.144 |
| **SDS BMI** |  |  |  | -1.19 | -1.78 – -0.60 | **<0.001** | -1.18 | -1.77 – -0.59 | **<0.001** |  |  |  |
| **AD ig gradient ENMO 0 24hr** |  |  |  |  |  |  | 4.79 | -2.81 – 12.40 | 0.216 | 2.01 | -7.02 – 11.04 | 0.662 |
| **AD ig gradient ENMO 0 24hr × Sex [M]** |  |  |  |  |  |  |  |  |  | 9.54 | -2.17 – 21.25 | 0.110 |
| **Random Effects** | | | | | | | | | | | | |
| σ^2^ | 83.05 | | | 76.84 | | | 76.75 | | | 78.27 | | |
| τ_00_ | 8.73 _School_ | | | 6.54 _School_ | | | 6.56 _School_ | | | 8.72 _School_ | | |
| ICC | 0.10 | | | 0.08 | | | 0.08 | | | 0.10 | | |
| N | 17 _School_ | | | 17 _School_ | | | 17 _School_ | | | 17 _School_ | | |
| Observations | 520 | | | 505 | | | 505 | | | 519 | | |
| Marginal R^2^ / Conditional R^2^ | 0.092 / 0.179 | | | 0.188 / 0.252 | | | 0.191 / 0.254 | | | 0.159 / 0.243 | | |
