# Supplementary material for: Average acceleration and intensity gradient of 9–11-year-old rural and urban Kenyan school-going children and associations with cardiorespiratory fitness and BMI: The Kenya-LINX project
Source: PLoS One. 2025 Aug 4;20(8):e0329173. doi: 10.1371/journal.pone.0329173 (PMC12321071; doi:10.1371/journal.pone.0329173)
Supplement: S2 Table — (DOCX) [file pone.0329173.s002.docx]

S2 Table. Association between BMI z-score and average acceleration

| **BMI and AvgAcc** | | | | | | | | | | | | |
| --- | --- | --- | --- | --- | --- | --- | --- | --- | --- | --- | --- | --- |
|  | *Model 1* | | | *Model 2* | | | *Model 3* | | | *Model 4* | | |
| **Predictors** | **Estimates** | **CI** | **p** | **Estimates** | **CI** | **p** | **Estimates** | **CI** | **p** | **Estimates** | **CI** | **p** |
| **(Intercept)** | 0.73 | 0.16 – 1.29 | **0.012** | 1.31 | -0.33 – 2.95 | 0.117 | 0.30 | -2.72 – 3.32 | 0.847 | 0.41 | -2.94 – 3.76 | 0.809 |
| **AD mean ENMO mg 0 24hr** | -0.02 | -0.03 – -0.01 | **<0.001** | -0.02 | -0.03 – -0.01 | **<0.001** | -0.02 | -0.03 – -0.01 | **0.001** | -0.02 | -0.03 – -0.01 | **0.001** |
| **Sex [M]** |  |  |  | 0.16 | -0.08 – 0.41 | 0.194 | 0.20 | -0.06 – 0.47 | 0.135 | -0.10 | -3.68 – 3.48 | 0.957 |
| **County [N]** |  |  |  | 1.01 | 0.70 – 1.32 | **<0.001** | 1.01 | 0.69 – 1.32 | **<0.001** | 1.01 | 0.69 – 1.32 | **<0.001** |
| **Age** |  |  |  | -0.10 | -0.24 – 0.04 | 0.155 | -0.10 | -0.24 – 0.03 | 0.139 | -0.10 | -0.24 – 0.03 | 0.141 |
| **AD ig gradient ENMO 0 24hr** |  |  |  |  |  |  | -0.44 | -1.55 – 0.66 | 0.431 | -0.38 | -1.70 – 0.93 | 0.567 |
| **AD ig gradient ENMO 0 24hr × Sex [M]** |  |  |  |  |  |  |  |  |  | -0.15 | -1.88 – 1.59 | 0.869 |
| **Random Effects** | | | | | | | | | | | | |
| σ^2^ | 1.72 | | | 1.73 | | | 1.73 | | | 1.73 | | |
| τ_00_ | 0.34 _School_ | | | 0.04 _School_ | | | 0.04 _School_ | | | 0.04 _School_ | | |
| ICC | 0.17 | | | 0.02 | | | 0.02 | | | 0.02 | | |
| N | 17 _School_ | | | 17 _School_ | | | 17 _School_ | | | 17 _School_ | | |
| Observations | 512 | | | 512 | | | 512 | | | 512 | | |
| Marginal R^2^ / Conditional R^2^ | 0.041 / 0.199 | | | 0.195 / 0.212 | | | 0.196 / 0.212 | | | 0.195 / 0.212 | | |
