# Supplementary material for: Average acceleration and intensity gradient of 9–11-year-old rural and urban Kenyan school-going children and associations with cardiorespiratory fitness and BMI: The Kenya-LINX project
Source: PLoS One. 2025 Aug 4;20(8):e0329173. doi: 10.1371/journal.pone.0329173 (PMC12321071; doi:10.1371/journal.pone.0329173)
Supplement: S3 Table — (DOCX) [file pone.0329173.s003.docx]

S3 Table. Association between cardiorespiratory fitness and intensity gradient

| **CRF and IG** | | | | | | | | | | | | |
| --- | --- | --- | --- | --- | --- | --- | --- | --- | --- | --- | --- | --- |
|  | *Model 1* | | | *Model 2* | | | *Model 3* | | | *Model 4* | | |
| **Predictors** | **Estimates** | **CI** | **p** | **Estimates** | **CI** | **p** | **Estimates** | **CI** | **p** | **Estimates** | **CI** | **p** |
| **(Intercept)** | 56.77 | 44.94 – 68.61 | **<0.001** | 27.53 | 10.50 – 44.55 | **0.002** | 12.69 | -8.29 – 33.67 | 0.235 | 5.90 | -17.17 – 28.98 | 0.616 |
| **AD ig gradient ENMO 0 24hr** | 18.93 | 13.27 – 24.59 | **<0.001** | 9.81 | 3.45 – 16.17 | **0.003** | 4.79 | -2.81 – 12.40 | 0.216 | 1.41 | -7.57 – 10.39 | 0.757 |
| **Sex [M]** |  |  |  | 4.62 | 2.81 – 6.42 | **<0.001** | 4.44 | 2.64 – 6.24 | **<0.001** | 21.52 | -2.67 – 45.71 | 0.081 |
| **County [N]** |  |  |  | -1.60 | -4.88 – 1.69 | 0.339 | -1.29 | -4.38 – 1.80 | 0.412 | -1.34 | -4.47 – 1.79 | 0.402 |
| **Age** |  |  |  | 0.79 | -0.18 – 1.75 | 0.109 | 0.74 | -0.22 – 1.70 | 0.129 | 0.74 | -0.22 – 1.70 | 0.132 |
| **SDS BMI** |  |  |  | -1.28 | -1.86 – -0.69 | **<0.001** | -1.18 | -1.77 – -0.59 | **<0.001** | -1.17 | -1.76 – -0.58 | **<0.001** |
| **AD mean ENMO mg 0 24hr** |  |  |  |  |  |  | 0.10 | 0.02 – 0.17 | **0.018** | 0.09 | 0.01 – 0.17 | **0.031** |
| **AD ig gradient ENMO 0 24hr × Sex [M]** |  |  |  |  |  |  |  |  |  | 8.28 | -3.41 – 19.97 | 0.165 |
| **Random Effects** | | | | | | | | | | | | |
| σ^2^ | 82.66 | | | 77.17 | | | 76.75 | | | 76.54 | | |
| τ_00_ | 11.51 _School_ | | | 7.89 _School_ | | | 6.56 _School_ | | | 6.82 _School_ | | |
| ICC | 0.12 | | | 0.09 | | | 0.08 | | | 0.08 | | |
| N | 17 _School_ | | | 17 _School_ | | | 17 _School_ | | | 17 _School_ | | |
| Observations | 520 | | | 505 | | | 505 | | | 505 | | |
| Marginal R^2^ / Conditional R^2^ | 0.083 / 0.195 | | | 0.176 / 0.253 | | | 0.191 / 0.254 | | | 0.192 / 0.258 | | |
