# Supplementary material for: Average acceleration and intensity gradient of 9–11-year-old rural and urban Kenyan school-going children and associations with cardiorespiratory fitness and BMI: The Kenya-LINX project
Source: PLoS One. 2025 Aug 4;20(8):e0329173. doi: 10.1371/journal.pone.0329173 (PMC12321071; doi:10.1371/journal.pone.0329173)
Supplement: S4 Table — (DOCX) [file pone.0329173.s004.docx]

S4 Table. Association between BMI z-score and intensity gradient

|  | | | | | | | | | |
| --- | --- | --- | --- | --- | --- | --- | --- | --- | --- |
|  | *Model 1* | | | *Model 2* | | | *Model 3* | | |
| **Predictors** | **Estimates** | **CI** | **p** | **Estimates** | **CI** | **p** | **Estimates** | **CI** | **p** |
| **(Intercept)** | -2.96 | -4.71 – -1.21 | **0.001** | -2.94 | -5.39 – -0.49 | **0.019** | 0.30 | -2.72 – 3.32 | 0.847 |
| **AD ig gradient ENMO 0 24hr** | -1.27 | -2.11 – -0.44 | **0.003** | -1.52 | -2.44 – -0.60 | **0.001** | -0.44 | -1.55 – 0.66 | 0.431 |
| **Sex [M]** |  |  |  | 0.17 | -0.10 – 0.43 | 0.222 | 0.20 | -0.06 – 0.47 | 0.135 |
| **County [N]** |  |  |  | 1.09 | 0.74 – 1.44 | **<0.001** | 1.01 | 0.69 – 1.32 | **<0.001** |
| **Age** |  |  |  | -0.11 | -0.25 – 0.03 | 0.136 | -0.10 | -0.24 – 0.03 | 0.139 |
| **AD mean ENMO mg 0 24hr** |  |  |  |  |  |  | -0.02 | -0.03 – -0.01 | **0.001** |
| **Random Effects** | | | | | | | | | |
| σ^2^ | 1.75 | | | 1.75 | | | 1.73 | | |
| τ_00_ | 0.42 _School_ | | | 0.06 _School_ | | | 0.04 _School_ | | |
| ICC | 0.19 | | | 0.03 | | | 0.02 | | |
| N | 17 _School_ | | | 17 _School_ | | | 17 _School_ | | |
| Observations | 512 | | | 512 | | | 512 | | |
| Marginal R^2^ / Conditional R^2^ | 0.018 / 0.206 | | | 0.173 / 0.201 | | | 0.196 / 0.212 | | |
