# Supplementary material for: Average acceleration and intensity gradient of 9–11-year-old rural and urban Kenyan school-going children and associations with cardiorespiratory fitness and BMI: The Kenya-LINX project
Source: PLoS One. 2025 Aug 4;20(8):e0329173. doi: 10.1371/journal.pone.0329173 (PMC12321071; doi:10.1371/journal.pone.0329173)
Supplement: S5 Table — (DOCX) [file pone.0329173.s005.docx]

S5 Table. VIF values for all models (Cardiorespiratory fitness and BMI z-score)

| **Variable** | **CRF**  **Model 1 (Unadjusted)** | **CRF**  **Model 2 (Adjusted)** | | **CRF**  **Model 3 (Adjusted)** | **CRF**  **Model 4 (Interaction)** | **BMI z-score Model 1 (Unadjusted)** | **BMI z-score Model 2 (Adjusted)** | **BMI z-score Model 3 (Adjusted)** |
| --- | --- | --- | --- | --- | --- | --- | --- | --- |
| Constant | 1.000 | | 330.134 | 448.243 | 472.091 | 1.000 | 330.134 | 448.243 |
| AvgAcc_ centered | 1.000 | | 1.011 | 1.033 | 1.034 | 1.000 | 1.011 | 1.033 |
| IG_ centered | 1.000 | | 1.011 | 1.042 | 1.189 | 1.000 | 1.011 | 1.042 |
| Age | - | | 1.157 | 1.206 | 1.207 | - | 1.157 | 1.206 |
| Sex | - | | 1.017 | 1.025 | 1.184 | - | 1.017 | 1.025 |
| Area of residence | - | | 1.066 | 1.077 | 1.078 | - | 1.066 | 1.077 |
| BMI z-score | - | | - | 1.139 | 1.139 | - | - | - |
| IG x Sex | - | | - | - | 1.272 | - | - | - |
